# Uncertainty and self‐efficacy in parents of a child with congenital cataract—New implications for clinical practice

**DOI:** 10.1002/nop2.256

**Published:** 2019-04-02

**Authors:** Jenny Gyllén, Gunilla Magnusson, Anna Forsberg

**Affiliations:** ^1^ Department of Clinical Neuroscience Institute of Neuroscience and Physiology Sahlgrenska Academy University of Gothenburg Gothenburg Sweden; ^2^ Region Västra Götaland, Sahlgrenska University Hospital Department of Ophthalmology Mölndal Sweden; ^3^ Care in High Tech Environments, Institute of Health Sciences at Lund University Lund Sweden; ^4^ Thoracic Unit Skåne University Hospital Lund Sweden

**Keywords:** congenital cataract, content analysis, nursing, parental perspective, qualitative, self‐efficacy, self‐management, uncertainty

## Abstract

**Aim:**

The aim was an in‐depth exploration of uncertainty and self‐efficacy among parents of a child with congenital cataract by means of two theoretical frameworks to re‐design family care.

**Design:**

A directed content analysis in accordance with Hsieh & Shannon, using Mishel's theory of uncertainty and Bandura's self‐efficacy theory.

**Methods:**

Open‐ended, in‐depth interviews were conducted with 23 parents of a child with congenital cataract; six mothers, five fathers and six couples.

**Results:**

In this novel study, self‐efficacy was interpreted as the ability to balance between uncertainty and acceptance. The performance accomplishment of the child and parents bridges the gap between uncertainty and acceptance by reducing uncertainty, thus constituting the level of self‐efficacy. Setbacks and complications increase uncertainty and reduce self‐efficacy, thus performance accomplishment is a mediator of self‐efficacy, while ability to master uncertainty determines the level of self‐efficacy.

## 1 INTRODUCTION

The rationale behind this study is that being a parent to a child with congenital cataract demands very good self‐management skills. Impact statements are listed in Table 1. Children diagnosed with a cataract comprise a heterogeneous group, ranging from healthy children with minimal visual impairment to children with multiple disabilities who require immediate surgery and, in many cases, re‐operations (Table 2). It is essential for the visual outcome that parents understand the purpose of amblyopia exercises (Drews‐Botsch, Celano, Kruger, & Hartmann, 2012; Handa & Chia, 2018; Li, Wang, & Xue, 2018; Singh et al., 2017; Timms, 2013). A previous study of the information needs of families that have a child with a cataract by Gyllén et al. (2015) shows that 72% of the families felt they did not receive sufficient information. Self‐efficacy is an important aspect of self‐management, which has gradually become the leading paradigm for long‐term management of chronic illness and thus highly relevant in this study. From research in other medical contexts, we know that uncertainty is an important aspect that diminishes self‐efficacy (Almgren, Lennerling, Lundmark, & Forsberg, 2017). However, little is known about the in‐depth meaning of uncertainty and self‐efficacy in this population, which hampers the possibility of empowering the parents to become their child's caregiver. Could it be that the parents’ need for information (Gyllén et al., 2015; Rahi, Manaras, Tuomainen, & Hundt, 2004; Rahi, Manaras, Tuomainen, & Lewando Hundt, 2005; Speedwell, Stanton, & Nischal, 2003) is in fact a marker of high levels of uncertainty and a low sense of self‐efficacy? Further research employing a novel approach is needed. Therefore, the aim of this study was an in‐depth exploration of uncertainty and self‐efficacy among parents of a child with congenital cataract by means of two theoretical frameworks to re‐design family care.

**Table 1:** Impact statements

The inside perspective on parental self‐efficacy demands a new ontological point of departure when discussing self‐management
Self‐efficacy from the parents’ perspective in the context of a child with congenital cataract is interpreted as finding a balance between uncertainty and acceptance, that is, finding the optimum level of self‐efficacy
The gap between uncertainty and acceptance is bridged by the performance accomplishment of the child and parents
The understanding of the impact of parental uncertainty and acceptance in combination with performance accomplishment indicates the need to redesign family care in cases of a child with congenital cataract

**Table 2:** Paediatric cataract

Paediatric cataract is a rare condition and about 40 such children are born per year in Sweden (Abrahamsson, Magnusson, Sjostrom, Popovic, & Sjostrand, 1999). The ophthalmology team expects the parents to take responsibility for the care after surgery, including the handling of contact lenses, patching, numerous visits to the clinic and managing everyday life with new prerequisites, which requires good self‐management skills on the part of the parents. Surgery is often followed by laborious visual training that continues for several years, during which parental cooperation is crucial (Timms, 2013). Previous studies show that patching is complex and parents experience it as stressful and hard work (Dixon‐Woods et al., 2006; Drews‐Botsch, Hartmann et al., 2012; Koklanis, Abel, & Aroni, 2006). Adherence to patching treatment is uncertain, leading to the following key questions; how can we understand parental adherence? and Is it possible to enhance parental adherence to patching? These two questions are the foundation of this study. Research reveals that less than half of all children who have undergone bilateral surgery in western Sweden achieved sufficient visual acuity to obtain a driving licence (Magnusson, Abrahamsson, & Sjostrand, 2002). For unilateral cataracts, good visual acuity (0.2 or more) was only achieved in children who underwent cataract surgery before the age of three months and who adhered to the occlusion therapy schedule (Allen, Speedwell, & Russell‐Eggitt, 2010; Lundvall & Kugelberg, 2002)

### 1.1 Theoretical framework

The middle‐range theory of uncertainty developed by Mishel (1990) was used to explain and comprehensively understand the phenomenon of uncertainty. During an illness, uncertainty may be a state where a person can experience a transition to a new perspective on life with a higher order and more complex orientation, enabling re‐organisation of self‐structure. Striving for control and predictability is a way for a person to achieve and maintain order and coherence (Mishel, 1990). In medicine, there is an implicit expectation that the cause of an illness can be determined with certainty and that the illness can be controlled (Mishel, 1990). However, there is a need for development of probabilistic and conditional thinking to create a new orientation towards life, including abandoning the expectation of continual certainty and predictability (Mishel, 1990). According to Mishel, by adopting such probabilistic thinking those coping with a chronic condition might accept that there are many options and opportunities in life that they can choose to focus on. This creates a need to redefine what is important in life, as well as learning to appreciate and accept the fragility and impermanence of life, which in time leads to a more balanced and stable existence (Mishel, 1990).

Self‐efficacy is a complex phenomenon introduced by Bandura (1997). In this study, parental self‐efficacy is defined as the parent's belief in her/his ability to succeed in managing the care after surgery. The efficacy expectations are based on four major components; performance accomplishments; vicarious experience, verbal persuasion and emotional arousal. These components are used by the person to evaluate her/his ability to succeed, that is, affecting her/his self‐efficacy but are in turn affected by contextual factors including social, situational and temporal aspects (Bandura, 1997).

For parents with children with a cataract, self‐management and self‐efficacy are highly relevant. How does uncertainty affect parental self‐efficacy in this population? To re‐design family care, the aim of this study was an in‐depth exploration of uncertainty and self‐efficacy among parents of a child with congenital cataract by means of two theoretical frameworks.

## 2 METHODS

### 2.1 Design

A directed content analysis (Hsieh & Shannon, 2005) using Mishel's theory of uncertainty and Bandura’s (1997) self‐efficacy theory was conducted retrospectively to deductively explore uncertainty and self‐efficacy. The rationale behind the choice of design was that the interviews were initially performed, analysed and presented elsewhere in accordance with constructivist grounded theory. During the naïve reading of the interviews and the analysis, it became obvious that the data were very rich and contained comprehensive descriptions of both experienced uncertainty and various self‐efficacy strategies that required an additional analysis.

### 2.2 Informants

The inclusion criteria were Swedish speaking parents whose child was born with congenital cataract that had been operated on, who visited the paediatric ophthalmological clinic in 2016–2017 and who were registered in the national quality register PECARE Exclusion criteria were not having other systemic co‐comorbidities. The parents came from Sweden or another European country (Holland, Poland, the UK, Serbia and Croatia). The nurse at the paediatric ophthalmology clinic contacted the potential informants and their written consent was obtained. They were informed that they could drop out of the study at any time. The informants were included consecutively, and 23 parents were interviewed. Demographic characteristics are presented in Table 3. About half of the children suffered from postoperative complications that required further surgery and most children had undergone patching treatment.

**Table 3:** Demographic characteristics

Nr	Parent	Age	Age	Child	Laterality	Age at diagnosis	Age at interview	Location of interview	Parent had cataract
1	Mother	39		Daughter	Bilateral	6 months	1.5 years	Hospital	No
2	Mother	42		Daughter	Unilateral	1 week	8 years	Hospital	No
3	Mother	41		Son(s)	Bilateral (both)	1–2 years	5–7 years	Hospital	Yes
4	Couple	39	41	Son	Bilateral	1 week	10 years	At home	No
5	Father	33		Son	Unilateral	6 months	2 years	At work	No
6	Father	33		Daughter	Bilateral	1 week	6 years	Cafe	Yes
7	Father	38		Daughter	Unilateral	2 months	7 years	Telephone	No
8	Father	46		Daughter	Unilateral	1 week	9 years	Telephone	No
9	Father	34		Daughter	Unilateral	1 week	4 years	Hospital	No
10	Couple	42	42	Son	Bilateral	1 week	11 years	At home	No
11	Couple	40	41	Daughter	Bilateral	4–5 years	10 years	At home	No
12	Couple	45	51	Daughter	Bilateral	1 week	8 years	At home	Yes
13	Couple	37	38	Daughter	Unilateral	1 week	4 years	At home	No
14	Mother	38		Son	Unilateral	1–2 months	8 years	Library	No
15	Mother	39		Son	Unilateral	1 week	4 years	At home	No
16	Mother	37		Daughter	Unilateral	1 week	5 years	At work	No
17	Couple	33	32	Son	Unilateral	1–2 months	3 years	At home	No

### 2.3 Data collection

Data collection took place between 2016–2017 in the form of interviews at one of the two hospitals in Sweden appointed to perform National Specialised Medical Care of children with congenital cataract. The open‐ended interviews that were re‐analysed had a mean duration of 52 min (22–87 min), resulting in approximately 300 pages of transcribed text.

### 2.4 Ethical considerations

Approval was obtained from the Regional Ethical Review Board in Gothenburg (no. 746‐14). The researcher (JG) who conducted the interviews had no relationship to the informants. A social worker at the follow‐up clinic was on hand to provide emotional support to the parents in the event that the interview would prove emotionally demanding for them. However, this need did not arise.

### 2.5 Data analysis

Analysis was performed according to the following steps:

1. Bandura's and Mishel's theory was scrutinised in detail.
2. We chose the main components of self‐efficacy and contextual factors from the theory and applied them to the data.
3. Two of the authors independently identified meaning units pertaining to uncertainty and self‐efficacy that corresponded to each of the chosen self‐efficacy components and factors.
4. The meaning units were compared and condensed in collaboration between the two authors.
5. All authors discussed the condensation and decided on which quotations should be presented.
6. All authors discussed the core meaning of uncertainty and self‐efficacy among parents of a child with congenital cataract.

### 2.6 Rigour

As authors, we found the directed content analysis challenging because we approached the data with an informed but strong bias (Hsieh & Shannon, 2005). We had previous knowledge of researching both uncertainty and self‐efficacy in a different medical context, which constituted a certain pre‐understanding. The concept of self‐efficacy is fairly complex compared with the more generic concept of uncertainty and in our opinion, the use of theoretical frameworks is necessary to capture its essence. To increase the trustworthiness of the analysis, the authors initially took a broad perspective aimed at capturing all possible occurrences of both uncertainty and self‐efficacy (Hsieh & Shannon, 2005). To avoid bias, we reflected jointly and individually when categorizing and scrutinizing the result, as well as attempting to remain as open as possible about our pre‐understanding and experience. We believe our results are transferrable to families of children with congenital cataract as well as to families of children with other visual impairments. To ensure further trustworthiness, we reflected on the concepts of credibility, dependability, conformability and transformability as described by Polit and Beck (2017).

## 3 FINDINGS

### 3.1 Part I: Uncertainty

Being a parent to a child with cataract means being in uncertainty from the moment the child is diagnosed until a state of acceptance is achieved. Parents who had experienced cataract themselves as children were more prepared for the diagnosis and experienced less uncertainty throughout the whole treatment process:

When it comes to the cataract it is not a big challenge for me because I grew up with a cataract. (Mother of two sons diagnosed at the age of 1–2 years)

For those who completely lacked knowledge and experience of childhood cataracts, receiving the diagnosis, often within the first postnatal week, was experienced as a great shock:

Cataract has never been part of my world, so it was a slight shock at that moment. (Mother of daughter diagnosed at the age of 6 months)

Uncertainty was triggered by the vulnerability of having to rely on the ophthalmological team and in particular on one expert physician, while the knowledge deficit led to a profound sense of lacking parental autonomy:

The thing with not knowing. Should we have the contact lens in when we come to the clinic? Eye‐patches on? What will happen first? After asking about 30 times, they finally said ‐ lens in at all times and patch always on. (Mother of daughter diagnosed during the first week)

**Figure 1:**
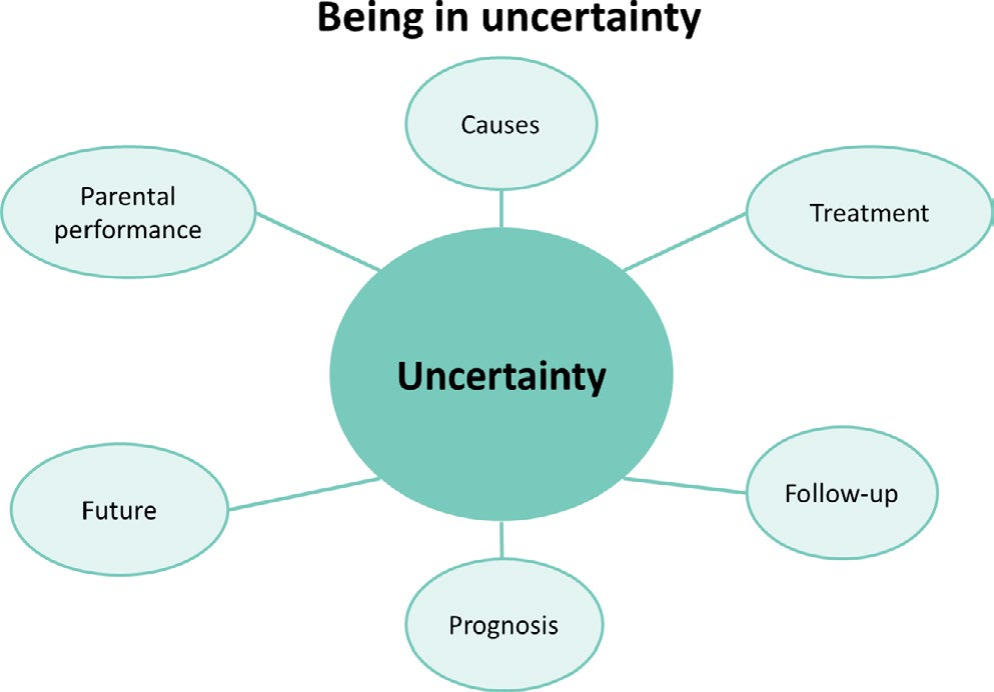
As visualized in figure, parental uncertainty involves many different aspects illustrated by several crucial questions: Where does the cataract stem from? Did I cause the cataract? (Causes) Is the occlusion therapy working? (Treatment) What will happen to my child? What is the plan for my child? (Follow‐up) Will my child be visually impaired or even blind for the rest of her/his life? (Prognosis) Will my child be able to drive a car, work and move out as an adult? (Future) Am I doing the right thing and am I doing enough as a parent? (Parental performance)

As visualized in Figure 1 parental uncertainty involves many different aspects illustrated by several crucial questions.

One part of uncertainty concerns the reason behind the disease and whether it is caused by the mother during pregnancy, resulting in self‐blame and guilt:

I know I had a cold and was prescribed penicillin in my 15th week. Is it some medicine I took during my pregnancy One blames oneself. (Mother of a son diagnosed in the first week after birth)

Being in uncertainty often meant being in despair and some parents experienced a profound sense of loneliness due to lack of support, leading to a wish to give up the occlusion therapy:

I often felt that I missed my son’s first year. He was so easy to take care of, but we never enjoyed him then…we shut ourselves away for, lets say, one year! (Mother of a son diagnosed one week after birth)

As a consequence, the parents developed a strong will to comprehend by looking for signs and cues to prevent or deal with various complications. Uncertainty was triggered by changes in treatment or suffering setbacks that gave rise to doubts about their own performance accomplishment. Every re‐operation, setback, side effect or complication from the treatment throws the parents back into uncertainty if they lack previous experience of cataracts and the ability to adjust their expectations. In a worst‐case scenario, their world is torn apart and their self‐efficacy is at zero:

It was getting close to the day for the operation; When we arrived the anaesthetist told us that there would be no operation because he had a cold. At that point my whole world came crashing down (Mother of a son diagnosed with a cataract at 18 months)

However, the motivation to continue with the occlusion and other parts of the treatment is strengthened by the wish to do the utmost for one's child:

For me it was a bit like, this is what you have to do, something similar to changing nappies. You don’t ask why you have to change nappies, you just do it… (Father of a daughter diagnosed in the first week after birth)

**Figure 2:**
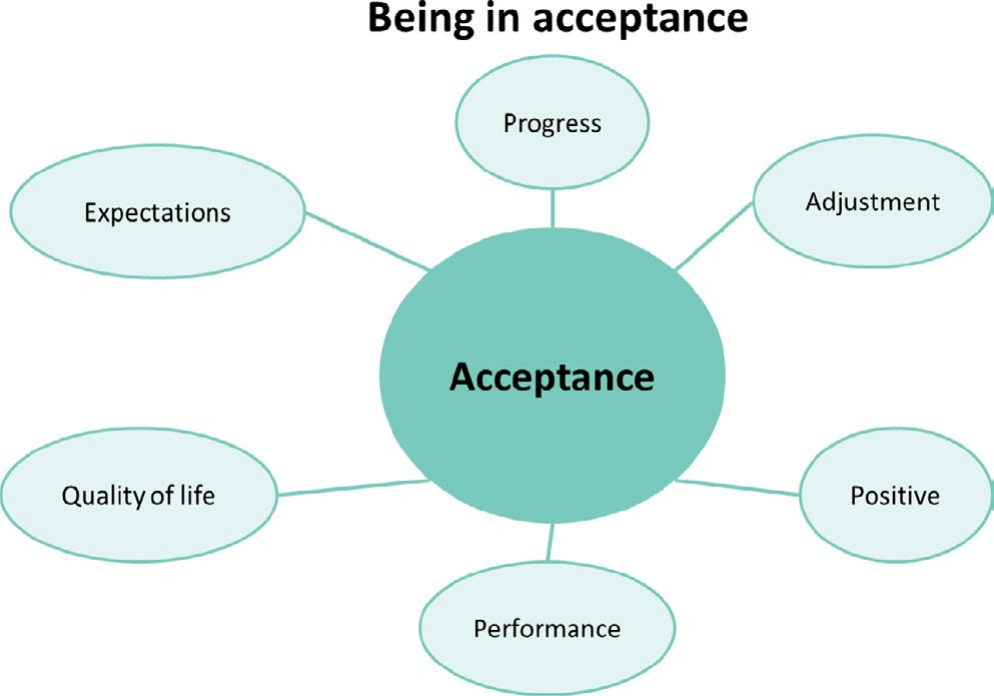
As visualized in figure, parental acceptance involves many different aspects illustrated by several areas in life

Self‐efficacy means balancing between uncertainty and acceptance. The state of acceptance occurs when uncertainty is reduced (Figure 2). For parents, being in acceptance means accepting the facts, observing the child's progress and enjoying the situation when the treatment works. Acceptance is enhanced when feeling optimistic, keeping one's perspective focused and acknowledging the fact that everyday life is actually working. Parents who are in acceptance look forward, focus on health‐related quality of life (HRQoL), decide not to worry too much about the future and adjust their expectations, thus managing to balance the child's abilities and inabilities.

### 3.2 Part II: Self‐efficacy

#### 3.2.1 Performance accomplishment

The single most prominent mediator of acceptance was performance accomplishment, which is also an important component of self‐efficacy and was evident in physical, mental, social and habitual aspects. We have chosen to merge these aspects instead of describing them separately, as caring for a child with congenital cataract means that they are interwoven. No complications and the absence of pain in the eye are considered to indicate progress:

I was so happy when he started to crawl from room to room on his own with his eye patch. He can see. It was great to receive confirmation that our hard work was paying off. It gave me a lot of encouragement and energy to keep on going. (Mother of a son diagnosed one week after birth)

Although they constantly focused on the child the parents could sometimes notice progress by their ability to adjust their expectations and enjoy the here and now:

We are extremely grateful it is only one eye!It is great to know that you can manage. I think it has to do with attitude. (Mother of a daughter diagnosed in the first week after birth)

#### 3.2.2 Complications, setbacks, treatment and side effects

Several physical complications and setbacks negatively affected the child's performance accomplishments and subsequently increased uncertainty:

We had to change the date for his christening more than once because he was scheduled for operations and we could not take the risk of him getting an infection afterwards. (Mother of a son diagnosed in the first week after birth)

The parental despair could be huge due to the struggle with the occlusion therapy and the knowledge deficit regarding what to expect or how to manage the child's frustration and anger:

We survived from complication to complication. We could not catch our breath before we had to cope with the next one. (Mother of a son diagnosed in the first week after birth)

#### 3.2.3 Vicarious experience

Vicarious experience means either being inspired or feeling downhearted as a result of hearing about other families’ experiences. Hearing about the negative experiences of other families or meeting families where the child suffers from a different and sometimes more disabling illness may lead to stress and constitute a reminder of all the possible complications that could occur:

We actually felt disheartened afterwards and hoped that the future would not continue to be as challenging for him as for the others. (Mother of a son diagnosed in the first week after birth)

A third form of vicarious experience is comparing one's situation with that of others and concluding that they are worse off in many ways:

If you look at others who have no legs, no arms and cannot walk you know they are doing a fantastic job. You have a minor visual impairment to cope with. Good Lord that’s nothing. (Father of a daughter diagnosed in the first week after birth)

#### 3.2.4 Verbal persuasion

The ophthalmological team provides structure by explaining the causes behind the cataract, the treatment and possible outcomes:

It is mostly because of X [physician]. He was calm, pedagogic and one felt safe. He radiated serenity so we dived right in, we can fix this. (Father of a daughter diagnosed in the first week after birth)

Verbal persuasion from the team can mediate positive thinking and constructive mastering strategies. It includes encouraging remarks about the occlusion therapy and various tips and tricks regarding how to manage the treatment at home. When the expert physician stresses the importance of adopting the right approach the parents listen and are inspired to adhere to the advice:

We always did as we were told, always! (Father of a son diagnosed in the first week after birth)

However, lack of structure on the part of the ophthalmology team, for example, lack of information, guidance and support, causes greater uncertainty and disappointment. Self‐efficacy then decreases and the parents have to find new mastering strategies to re‐enter the acceptance phase:

When we went for one of her follow‐ups at the clinic we were asked, "How many hours do you use the eye patch"? We were shocked. We had received instructions so we counted the hours and the minutes. I lost trust in the clinic when we were asked how long we kept the eye patch on as they were the ones giving us the instructions. I have no medical training! I refuse to listen to such questions. **I** should be able to ask the questions and get support. (Mother of a daughter diagnosed in the first week after birth)

#### 3.2.5 Emotional arousal and outcome expectations

Several situations led to great emotional arousal. The first is the time of diagnosis, which as described earlier, causes immediate concern and sometimes shock. A second extremely emotional situation mentioned by all parents is handing over one's child for anaesthesia and surgery:

There is nothing worse than seeing your child under anaesthesia… (Father of a daughter diagnosed at the age of 4.5 years)

However, the most profound cause of negative emotional arousal is the constant battle with the child regarding the occlusion therapy. There is a strong sense of disappointment when visual achievements are lacking and accomplishments are poor in terms of the child's ability to accept the therapy. The parents’ willpower weakens when there are no obvious improvements in health and well‐being. They consider that their efforts are in vain, which gives rise to depressive thoughts. Fatigue due to lack of sleep, lack of support from relatives and fear that if they give up the child will become blind cause negative emotions:

…if I don't fight he will be blind in one eye. It's up to me and that is all there is to it. (Mother of a son diagnosed in the first week after birth)

Not all families experience this struggle, as some parents adopt creative strategies that work well. Our study revealed a clear pattern where parents with a high level of uncertainty and subsequently low self‐efficacy found it harder to manage the occlusion therapy, which led to great sadness and despair:

When we put the patch on he blew his top, he was furious. It got so bad that we went to the hospital and were there for two days so that he could play while getting used to the patches. This helped us to get back on track again. (Mother of a son diagnosed at the age of 1.5 years)

#### 3.2.6 Performance adjustment

Parents who manage the whole process, in particular the occlusion therapy, have a unique ability to adjust their mastering strategies, performance expectations and perspective on their child, which implies that they employ self‐efficacy:

You could say that you don't identify yourself as the parent of a disabled child. (Father of a daughter diagnosed in the first week after birth)

Parents with high self‐efficacy manage to set goals, keep them visible and focus on them:

It would be great if she could see well enough to get her driving license with the impaired eye just in case something happens to her healthy eye. It is a motivation for both of us. (Father of a daughter diagnosed in the first week after birth)

Support from the child's grandparents and/or pre‐school enables new routines and facilitates the child's treatment. Adaptation strategies involve meeting the performance accomplishment expectations of relatives, friends and healthcare professionals. Clear gender differences in how the parents adjust to the new life situation were evident both when they were interviewed separately and together as a couple. In general, fathers focus on the positive aspects, performance and goal setting:

It is probably a little more in the male nature that one has to have a goal. (Father of a daughter diagnosed in the first week after birth)

In contrast, mothers often assume the role of caregiver, taking the main responsibility for the occlusion treatment and check‐ups at the clinic. In a worst‐case scenario, this can lead to a disturbed relationship with the child, requiring professional counselling to break the viscous circle:

I did not see my daughter as my child for four years. I saw her medical and physical needs. In that situation it is actually very easy for a parent to loose contact with the child. Our relationship did not click because she saw me as a doctor‐mother in the same way. When she was older we had no choice but to go to family therapy. (Mother of a daughter diagnosed in the first week after birth)

Even when the mothers adopted conscious strategies to focus on positive thoughts, they were generally more worried about the child's future than the fathers. For both mothers and fathers, keeping up one's spirits, not giving in, using will power and accepting the temporary role of being a care giver to their child created positive emotions.

## 4 DISCUSSION

To the best of our knowledge, this is the first study to focus on the parental perspective of living with a child with congenital cataract. It therefore provides a much needed insight into the struggle of parental care giving. The meaning of parental self‐efficacy for these parents is mastering the delicate balance between uncertainty and acceptance. Like a pendulum, the parents move between these two states in a never‐ending process of adjustment in their everyday family life. Uncertainty arises the moment the parents have to face the fact that their child suffers from cataract. It is also triggered by the vulnerability of having to rely on the expertise of one single physician. Lack of information from members of the ophthalmological team causes uncertainty and a sense of being lost in a circle of trial and error. The parents’ knowledge deficit results in lack of autonomy and a need to comprehend by looking for signs and dealing with setbacks to reduce uncertainty. In turn, self‐efficacy is increased by the child's performance accomplishment, making it easier to be in acceptance regarding the child's health status and the subsequent impact on the family's daily life. Self‐efficacy seems to be triggered by observing the child's progress and emphasizing her/his performance accomplishment, which in turn leads to the ability to be pragmatic and relaxed, determined and optimistic, adjust expectations, focus on quality of life and look to the future.

However, the balance between uncertainty and acceptance is delicate and easily affected by changes in treatment, frequent setbacks and complications, as well as negative vicarious experiences from other families or on the Internet. Expert clinicians can guide the parents back to acceptance by providing structure, verbal persuasion and extensive guidance and support. Expert clinicians should also be fully aware of the fact that every setback is a step towards uncertainty and that every performance accomplishment is an important step towards self‐efficacy. Mastering this delicate balance is equally important for expert clinicians and parents involved in the care of their child with cataract.

It is obvious that parents with previous experience of cataracts are better equipped in terms of adjusting expectations and setting realistic goals. Fathers seem to prefer a salutogenic approach that involves prioritizing health, goalsetting and progress in contrast to mothers, who more often assume the role of primary care giver and subsequently worry about different aspects of the child's illness. This calls for the ophthalmological team to be more observant of mothers to recognise their concerns and encourage fathers to be more perceptive. The fathers’ salutogenic approach might be an asset. A key assumption in family nursing is that “a change in one family member affects all other family members” (Benzein, Hagberg, & Saveman, 2012; Wright & Leahey, 2013). This is true for both positive and negative things; in this population, it is applicable to the fathers’ salutogenic approach, which can be recognised and introduced to the mothers.

We have to understand uncertainty as a natural state that occurs when parents witness their child suffering from a condition that shatters their worldview. Events must be structured, ordered and predictable (Antonovsky, 1987). The sense of coherence is lost when the stimuli associated with illness, treatment and recovery are vague, ill‐defined, probabilistic, ambiguous and unpredictable (i.e. uncertain). “The uncertainty in the illness situation is the source of a flux that shifts the person from an original position through a point of bifurcation towards a new state” (Mishel, 1990). For the ophthalmology team to support this transition, it is essential to understand that uncertainty per se is not a negative emotion (Mishel, 1990). Instead, it must be seen as a medium that enables reorganization and makes life understandable (Mishel, 1990). Other factors that influence the parents’ formation of a new orientation in life are previous life experience, social resources and the attitude of healthcare professionals (Mishel, 1990), which are clearly evident in the findings.

In our opinion, the current treatment and follow‐up of children with congenital cataract are structured around medical aspects, visual ability and scanning for high ocular pressure or other complications. This means a risk that the ophthalmology team struggles to obtain medical certainty at the expense of increasing parents’ uncertainty. This new paradigm in the care of children with cataract requires reflection on the organization where the treatment takes place.

We argue that the parents’ uncertainties should be assessed regularly and that every family should be assigned a family case manager with a clear mission to pilot the parents and act as a coach. Research in this and other areas demonstrates that case managers are an important tool for parental support (Greco, Sloper, Webb, & Beecham, 2007; Ling, McCabe, Brent, & Crosland, 2013; Rahi et al., 2004; Sloper, Greco, Beecham, & Webb, 2006). Every setback and complication requires an educational conversation, starting with the concrete question: How do you perceive this situation? How do you understand this? Depending on the response, the case manager should provide individual structure. If the level of uncertainty is too high and consequently the self‐efficacy too low, there is an imminent risk of the parents giving up the occlusion therapy and use of lenses and developing a sense of complete parental failure. This is an undesirable consequence, as previous research shows that patching is crucial for the child's visual status (Charters, 2017; Dixon‐Woods, Awan, & Gottlob, 2006; Drews‐Botsch, Hartmann, & Celano, 2012; Lundvall & Kugelberg, 2002).

This study reveals that lay support and parental support groups may not be helpful, as other families’ negative experiences create uncertainty and diminish self‐efficacy. However, some parents in this study expressed a desire for more contact with other parents in the same situation, which corresponds with a finding in a study by Gyllén et al. (2015). The parents specifically requested tips for occlusion therapy, how to put the contact lens in etc. It seems as if the need for vicarious experience concerns practical issues, while hearing about other families’ struggle can have a negative effect, that is, create more uncertainty. The factors behind this require further investigation.

Adjustment is necessary to maintain a successful balance that leads to acceptance in terms of both performance and outcome expectations. Performance accomplishment is the component that had the greatest impact on self‐efficacy and acceptance. Adjustment can and should be supported and facilitated by the ophthalmology team. It is evident that even when parents adhere to the advice provided there is no guarantee that it will lead to acceptance and well‐being. The key to acceptance and well‐being is confirmation of the parents’ struggle, efforts and the child's progress by the ophthalmology team. Without this confirmation uncertainty may continue, followed by emotional arousal. As discussed by Almgren, Lennerling, Lundmark, & Forsberg (2017), our healthcare system has a strong focus on behaviour and performance, which means an implicit risk of being disappointed as a parent when one behaves in accordance with the advice provided (verbal persuasion) but the results are lacking. This creates feelings of uncertainty and a possible ineffective loop. Thus, a fundamental understanding of the balance between uncertainty and acceptance, that is, parental self‐efficacy, paves the way for extensive improvements in the care of these families. In clinical practice, there is a possibility that healthcare professionals confuse uncertainty and distress, interpreting parents’ mastering as lack of adherence. We argue that this study shows the need for healthcare professionals to examine whether there are sources of uncertainty rather than assuming that the problem is due to distress. The team must then guide the parents back towards acceptance, recognizing that uncertainty will always be present to some degree when in parents of a child with cataract. We conclude that most parental distress in this context actually stems from uncertainty, demanding a different approach from the ophthalmology team. Clinical implications are listed in Table 4.

**Table 4:** Clinical implications

1. Assessment of uncertainties and expectations
2. Provide careful verbal and written piloting from the start
3. Support and evaluate adjustment efforts
4. Confirm that uncertainty is a natural part of being a parent to a child with cataract
5. Appoint a family case manager

In conclusion, this study generated the following hypothesis:

- self‐efficacy from the parents’ perspective is about balancing between uncertainty and acceptance through performance accomplishments.
- balancing expectations might diminish disappointments, which could also reduce distress.
- fathers might be better equipped than mothers to adopt a salutogenic perspective due to less care giver burden.

### 4.1 Study limitations

Most of the informants were born in Sweden or another European country, which limits the transferability to a solely western perspective. We assume that the parents who accepted the invitation to participate were those who coped fairly well with the treatment process. In addition, there is always a risk of recall bias in a retrospective study. Finally, the findings are based on our interpretation of Mishel's theory and Bandura's theory, which other researchers might understand differently.

## ETHICAL APPROVAL

Ethical approval was obtained from the Regional Ethical Review Board in Gothenburg, Sweden (no. 746‐14).

## AUTHOR CONTRIBUTIONS

JG: performed all interviews, analysed data and wrote manuscript; GM: wrote the manuscript; AF: designed the study, analysed data and wrote the manuscript. All authors discussed the results and contributed to the final manuscript.
